# Anti-PSMA ^124^I-scFvD2B as a new immuno-PET tool for prostate cancer: preclinical proof of principle

**DOI:** 10.1186/s13046-019-1325-6

**Published:** 2019-07-23

**Authors:** B. Frigerio, S. Morlino, E. Luison, E. Seregni, A. Lorenzoni, A. Satta, R. Valdagni, A. Bogni, C. Chiesa, M. Mira, S. Canevari, A. Alessi, M. Figini

**Affiliations:** 1 Biomarkers Unit, Department of Applied Research and Technical Development, Fondazione IRCCS Istituto Nazionale dei Tumori, Milan, Italy; 2 Radiation Oncology 1, Fondazione IRCCS Istituto Nazionale dei Tumori, Milan, Italy; 3 Nuclear Medicine, Fondazione IRCCS Istituto Nazionale dei Tumori, Milan, Italy; 4 Fondazione IRCCS Istituto Nazionale dei Tumori, Milan, Italy; 5 Department of Oncology and Hemato-oncology, University of Milan, Prostate Cancer Program, Fondazione IRCCS Istituto Nazionale dei Tumori, Milan, Italy; 6 Present address: Fisica Sanitaria - ASST Ovest Milanese, Via Papa Giovanni Paolo II, Legnano, Milan Italy

**Keywords:** PCa, ^124^I, scFv, Antibody fragment, PET

## Abstract

**Background:**

Prostate cancer (PCa) is the second leading cause of cancer-related death in the Western population. The use in oncology of positron emission tomography/computed tomography (PET/CT) with emerging radiopharmaceuticals promises accurate staging of primary disease, restaging of recurrent disease and detection of metastatic lesions. Prostate-specific membrane antigen (PSMA) expression, directly related to androgen-independence, metastasis and progression, renders this tumour associate antigen a good target for the development of new radiopharmaceuticals for PET. Aim of this study was to demonstrate in a preclinical in vivo model (PSMA-positive versus PSMA-negative tumours) the targeting specificity and sensitivity of the anti-PSMA single-chain variable fragment (scFv) labelled with ^124^I.

**Methods:**

The ^124^I-labeling conditions of the antibody fragment scFvD2B were optimized and assessed for purity and immunoreactivity. The specificity of ^124^I-scFvD2B was tested in mice bearing PSMA-positive and PSMA-negative tumours to assess both ex-vivo biodistribution and immune-PET.

**Results:**

The uptake fraction of ^124^I-scFvD2B was very high on PSMA positive cells (range 75–91%) and highly specific and immuno-PET at the optimal time point, defined between 15 h and 24 h, provides a specific localization of lesions bearing the target antigen of interest (PSMA positive vs PSMA negative tumors %ID/g: *p* = 0.0198 and *p* = 0.0176 respectively) yielding a median target/background ratio around 30–40.

**Conclusions:**

Preclinical in vivo results of our immuno-PET reagent are highly promising. The target to background ratio is improved notably using PET compared to SPECT previously performed.

These data suggest that, upon clinical confirmation of sensitivity and specificity, our anti-PSMA ^124^I-scFvD2B may be superior to other diagnostic modalities for PCa. The possibility to combine in patients our ^124^I-scFvD2B in multi-modal systems, such as PET/CT, PET/MR and PET/SPECT/CT, will provide quantitative 3D tomographic images improving the knowledge of cancer biology and treatment.

## Background

Prostate cancer (PCa) is the most common tumor among males in Europe over the age of 70 [1]. The management of the patient with PCa is complex. It differs according to the patient’s age, comorbidities, stage and disease histology. Guidelines for clinical practice include active surveillance, watchful waiting, surgery, radiotherapy, androgen ablation [2]. Recent diagnostic advances have led to some developments in the management of PCa patients [3].

There is a need to plan more personalized observational or therapeutic approaches for PCa patients to both manage disease at first diagnosis and treat disease relapses. For all these strategies it is necessary to have non-invasive diagnostic investigations, which allow promptly and precisely identifying the position and number of lesions. Conventional anatomical imaging modalities initially used were ultrasound, computed tomography (CT), bone scintigraphy, and magnetic resonance imaging (MRI). These techniques may offer information on morphological tumors, but they have considerable limitations for staging and monitoring the disease, especially in patients with low PSA levels [4, 5]. Several studies on the use of CT and MRI produced unsatisfactory data for sensitivity to PCa metastasis detection [6]. Among imaging techniques, PET or PET/CT use of [^18^F]FDG is limited in PCa [7]; indeed, PCa glucose utilization is low, and FDG uptake is insufficient in up to 81% of primary tumors [8, 9]. Other metabolic tracers, such as [^11^C]Choline- or [^18^F]Fluorocholine, have shown some promising results in the clinic but choline uptake was detected not only in malignant but also in hyperplastic prostate tissues [10].

Immuno-imaging based on labelling of a mAb or mAb fragment with high specificity and high-affinity binding to a specific tumor associated antigen was then proposed and radiotracers based on the prostate specific membrane (PSMA) have been developed.

PSMA is a cellular membrane protein that has a significant increase in prostate cell expression compared to other tissues (kidney, small intestine and salivary glands). Its location is trans-membrane with a dominant extracellular proportion. The expression of PSMA is increased in many tumors [11] but its level is the highest in PCa where the expression is almost invariant between primary and metastatic lesions. Therefore PSMA was considered an excellent target for PCa imaging.

The ^111^In-labelled capromab pendetide (ProstaScint) is one of the first anti-PSMA monoclonal antibody radioconjugate that is useful for SPECT imaging [12]. A phase I imaging trial with ^89^Zr-labelled J591 in 10 patients with metastatic PCa also demonstrated the ability of this construct to identify metastases, including lesions that were not detected on conventional imaging [13]. In addition, a recent prospective study found that ^89^Zr- J591 in patients awaiting prostatectomy was able to identify tumours with a Gleason score of seven or greater [14]. Unfortunately major disadvantages have been described with these radiolabeled monoclonal antibodies such as the long time from injection for optimal imaging, with several days often required due to poor tumour penetration.

To offer greater diagnostic-therapeutic precision it is necessary to look for further developments. Antibody formats, such as single-chain antibodies [15], diabodies, nanobodies and minibodies have now been labeled with positron emitters, allowing rapid imaging with high contrast [16].

Against PSMA we developed a very promising monoclonal antibody and the derived scFvD2B able to localize tumours in different mouse models [17–20]. This scFv represents a valid tool as tracers for in vivo radio diagnostics thanks to its binding kinetic (fast association to the recognised antigen and slow dissociation) allowing a high accumulation of the antibody fragment at the tumour site, despite its monovalent binding. Moreover the short circulation half-life of the scFv represents an advantage over the entire antibody. The use of antibody fragments may accelerate the time required for diagnosis whilst maintaining tumours specificity. Tumours can be visualised more rapidly, and optimal images can be acquired within 24 h after injection avoiding irradiation of normal tissues. Aim of this study was to evaluate in vivo model the target specificity for PSMA expressing tumours by using ^124^I-scFvD2B.

## Methods

### Antibody and cell lines

The scFvD2B antibody fragment used in this study was previously described [19, 20]. PC3 and LNCaP (human PCa) and A431 (epidermoid carcinoma) cell lines were purchased from the American Type Culture Collection (ATCC; Manassas, MD). The PC3-PIP cell line, which is isogenic to PC3 and stably expresses PSMA [21] was kindly provided by Dr. W. Heston (Cleveland). Cell lines were subjected to short tandem repeat (STR) analysis in accordance with the ATCC guidelines, and the genetic profiles were compared to publically available databases to verify authenticity. The cell lines were maintained in vitro in RPMI 1640, 10% foetal bovine serum (FBS), and 2 mM glutamine at 37 °C in a humidified atmosphere of 5% CO_2_/95% air.

### ^124^I-scFvD2B radiolabeling

Radioiodination of scFvD2B with ^124^I (half-life: 4.176 days) was performed as following: Na ^124^I (Perkin Elmer), after oxidation in an iodogen-coated tube (Pierce, Rockford, IL) with 0.1 ml of iodination buffer (TRIS-HCl 25 mM, pH 7.4 + NaCl 0.4 M) for 5 min at room temperature, was added to scFvD2B for 10 min at room temperature with gentle rotation.

The radiolabeled reagent was purified using a PD-10 desalting column and eluted with Sodium Phosphate buffer 100 mM pH 7.4 + NaCl 150 mM + Ascorbic Acid 10 mg/ml. The fractions corresponding to the radiotracer peaks were pooled and counted into a COBRA II Auto-Gamma counter (Packard, PerkinElmer, Boston, MA). The labelling efficiency before gel filtration and the final radiochemical purity after purification, calculated as amount of radioactivity associated to the protein vs the fraction of free iodine, available after the reaction, were determined by instant thin-layer chromatography on silica gel strips (ITLC-SG; Gelman Sciences, Ann Arbor, MI) using Methanol/Physiologic Sodium Chloride solution (1:1, v/v), as the mobile phase, read by Cyclone Storage Phosphor System and analyzed by Optiquant Image Analysis Software (Packard).

### Immunoreactivity

The specific immunoreactivity of ^124^I-scFvD2B was assayed on 1 × 10^7^ freshly detached PSMA positive (PC3-PIP and LnCaP) and PSMA negative (PC3 and A431) cells incubated with trace amount of ^124^I-scFvD2B in 250 μl of 0.03% BSA in PBS (Phosphate Buffer Saline) for 3 h at room temperature with gentle rotation. Then, the cells were washed three times with cold medium. The activity in the supernatants and in the cell pellets was determined in the gamma counter and the immunoreactivity was calculated as follow: (cell pellet radioactivity/cell pellet and unbound radioactivity) × 100.

### Murine models

**Fig. 1:**
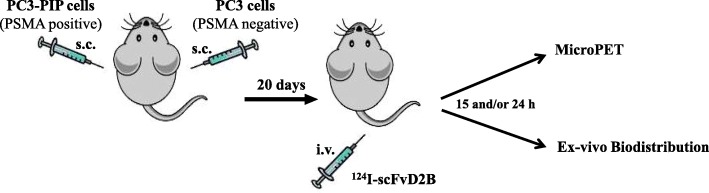
Cartoon illustrating the in vivo procedure: 5 × 10^6^ of either PC3-PIP (PSMA positive) or PC3 (PSMA negative) cells were injected subcutaneously (s.c.) in the flank of mice. 20 days later, ^124^I-scFvD2B was administered i.v. and after 15–24 h both ImmunoPET imaging and ex vivo biodistribution were performed

All protocols were approved by the Ethics Committee for Animal Experimentation of the Fondazione IRCCS Istituto Nazionale dei Tumori (internal code PROG-INT-12-2012) and communicated to the minister of health. Procedures involving animals and care were in conformity with institutional guidelines that comply with national and international laws and policies (D.L. 116/92 and subsequent implementing circulars). Male CD1 nude mice (6–7 weeks old) were purchased from Charles River Laboratories and acclimatized for at least 1 week before tumour cell injection. One volume (50 μl) of saline containing 5 × 10^6^ of either PC3-PIP or PC3 cells was mixed with 2 volumes of Matrigel (BD Biosciences) and injected subcutaneously in the flank. Tumour uptake was monitored over time and animals were administered with ^124^I-scFvD2B 20 days later (the mean tumour weight was 0.147 ± 0.07 g for PC3-PIP, 0.118 ± 0.03 g for PC3). For animal model protocol see Fig. 1. The 3R ethical rules for animal experiments were considered to give this proof of principle (https://www.ema.europa.eu/en/human-regulatory/research-development/ethical-use-animals-medicine-testing).

### In vivo immuno-PET and ex vivo biodistribution

Thyroid uptake of radioiodine was blocked by adding Lugol’s solution 0.02% in the drinking water 1 week before the injection of radiopharmaceutical and until the end of the experiment.

To have a proof of principle, as first, two mice bearing in one flank PSMA-positive and in the controlateral flank PSMA-negative tumours were injected via the tail vein with of 3.7 MBq of ^124^I-scFvD2B (corresponding to 22.2 μg of ScFvD2B). PET images were acquired 24 h after radiopharmaceutical injection (Table 1).

**Table 1:** Summary of radiochemical characteristics and bioactivities of scFvD2B after labeling with ^124^I

Radiolabeling experiments	1	2	3	4
% Efficiency	91	94	94	98
% Radiochemical purity	98	100	100	100
% Immunoreactivity on				
PSMA positive cells				
PC3-PIP	91	82	82	88
LNCaP	84	82	75	80
PSMA negative cells				
PC3	1.3	1.1	2.0	3.2
A431	0.9	0.2	0.4	0.4
Specific Activity (MBq/mg)	166.5	196.1	381.1	432.9

After in vitro optimization, a final in vivo experiment was performed using 5 mice bearing PSMA-positive and PSMA-negative tumours injected with 7.4 MBq of ^124^I-scFvD2B (corresponding to 17 μg of ScFvD2B, Table 1). PET images were acquired 15 and 24 h after radiotracers administration.

Animals were anesthetized using isoflurane and imaged with micro-PET eXplore VISTA GE Healthcare. Images were reconstructed with 3D FORE/2D OSEM algorithms and analyzed by eXplore VISTA software.

The mice bearing tumours were analyzed for ex vivo biodistribution of ^124^I-scFvD2B. At 15 or 24 h, animals were bleed and sacrificed to allow tissue of interest collection. Blood and organ samples were then wet-weighed and counted in a gamma counter with internal standard to correct the decay. Measurements were expressed as percentage of injected dose per gram of tissue (%ID/g) and as tissue/tumour to blood ratio (T/B). Statistical analysis was performed with GraphPad Prism® 5.02 version.

## Results

**Fig. 2:**
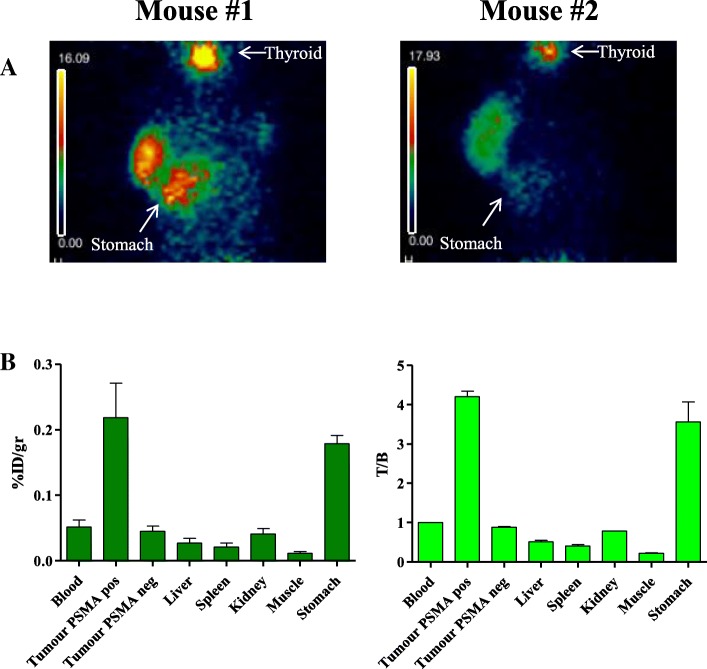
ImmunoPET imaging and ex vivo biodistribution analysis with ^124^I-scFvD2B of experiment #1: **a** Immuno-PET images after intravenous administration of 3.7 MBq ^124^I^−^scFvD2B (166.5 MBq/mg) in two mice. PC3 (PSMA negative, right flank) and PC3-PIP (PSMA positive, left flank) tumours evaluated 24 h post injection. Stomach and Thyroid are indicated by an arrow. The imaging is so clean in the irrelevant organ that it is barely visible the shape of the mice. **b** Ex vivo biodistribution analysis in PC3 and PC3-PIP tumours in the same two mice. Uptake and retention were measured in different organs and illustrated both as decay-adjusted percentage of the injected dose per gram of tissue (% ID/g) and T/B. The background activity observed in the stomach was probably due to the free ^124^I or the possible ingestion of the sawdust contaminated by the radioactive fraction present in the mice urine since was not present in the fourth experiment done in a larger number of animals (Fig. 3b)

High radiolabeling efficiency (range 91–98%) and radiochemical purity (range 98–100%) are obtained in 4 different radiolabeling experiments of scFvD2B with ^124^I (Table 1). On the basis of our previous work with ^123^I [20], the first labeling was performed in similar experimental conditions with a quite low ^124^I/protein ratio (166 MBq/mg) to preserve immunoreactivity (Exp #1, Table 1). ^124^I-scFvD2B was administered in animals bearing in one flank PSMA-positive and in the controlateral flank PSMA-negative tumours (PC3-PIP and PC3 cells, respectively). At 24 h post injection biodistribution demonstrated relevant uptake in PSMA-positive tumors, while the radiotracer’s uptake in PSMA negative tumors was similar to the distribution in non-target organs (%ID/g: Fig. 2a; T/B: Fig. 2b).

In order to obtain a higher T/B ratio other two in vitro radiolabeling experiments (Exp #2 and #3, Table 1) were performed increasing the specific activity; the immunoreactive fraction still remained very high on PSMA positive cells (range 75–91%) and highly specific, as shown by the negligible binding on PSMA negative cells.

**Fig. 3:**
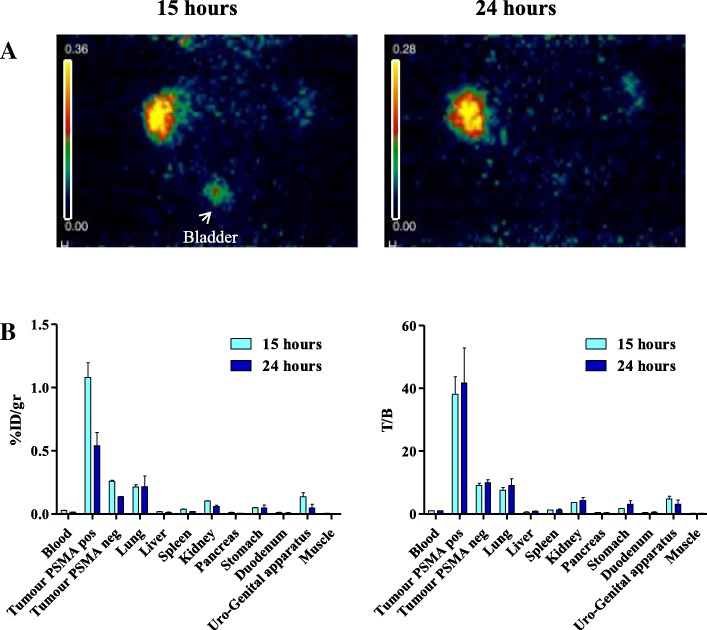
ImmunoPET imaging and ex vivo biodistribution analysis with ^124^I-scFvD2B of experiment #4. **a** One representative Immuno-PET images after intravenous administration of 7.4 MBq ^124^I^−^scFvD2B (433 MBq/mg). PC3 (PSMA negative, right flank) and PC3-PIP (PSMA positive, left flank) tumours evaluated 15 and 24 h post injection. Bladder is also indicated by an arrow. The imaging is so clean in the irrelevant organ that it is barely visible the shape of the mice. **b** Ex vivo biodistribution analysis in PC3 and PC3-PIP tumour bearing mice evaluated 15 h (2 mice) and 24 h (3 mice) post injection of ^124^I-scFvD2B. Uptake and retention were measured in different organs and illustrated both as decay-adjusted percentage of the injected dose per gram of tissue (% ID/g) and T/B

Based on these results, the radiolabeling experiment # 4 (433 MBq ^124^I for mg of protein) was used for biodistribution and immuno-PET analysis at 15 and 24 h post injection of ^124^I-scFvD2B. Thanks to the high specific activity and dose injected (7.4 MBq) the specific uptake confined to the PSMA-positive tumour both at 15 and 24 h improved, with low uptake in the PSMA-negative tumour. Immuno-PET analysis was performed in 5 animals and for two of them the acquisition was performed both at 15 and 24 h and one is displayed in Fig. 3a. Low background uptake was detected in the surrounding tissue, providing excellent contrast images, as confirmed by ex-vivo biodistribution data (Table 2). Overall, the data showed specific localization of the tracer; 15 h post injection the uptake in PSMA-positive tumors was higher than in blood, lungs, liver, and kidneys. On the contrary PSMA-negative tumors showed marginal ^124^I-scFvD2B uptake 15 h post injection (PSMA positive vs PSMA negative tumors %ID/g: *p* = 0.0198). After 24 h, ^124^I-scFvD2B was cleared from blood and kidneys, while the uptake in the PSMA-positive tumours remained still evident and around 4 times higher than in the PSMA-negative tumours (PSMA positive vs PSMA negative tumors %ID/g: *p* = 0.0176) (Fig. 3b).

**Table 2:** Summary of ex vivo biodistribution of ^124^I-scFvD2B in mice with subcutaneous (PSMA)-negative and PSMA-positive xenografts

Tissue/organ^a^	EXPERIMENT #4			
	15 h		24 h	
	Mean	SD	Mean	SD
Blood	0.028	0.001	0.014	0.003
Tumour pos	1.079	0.166	0.540	0.180
Tumour neg	0.258	0.014	0.135	0.008
Lungs	0.214	0.023	0.216	0.146
Liver	0.017	0.004	0.012	0.005
Spleen	0.036	0.004	0.018	0.003
Pancreas	0.103	0.004	0.059	0.019
Kidney	0.013	0.002	0.005	0.001
Stomach	0.049	0.002	0.047	0.040
Duodenum	0.013	0.003	0.008	0.003
Uro-genital apparatus	0.137	0.044	0.048	0.049
Muscle	0.006	0.000	0.003	0.002

^a^tissue uptake expressed as % ID/g; 2 animals were evaluated at 15 h and 3 animals at 24 h

## Discussion

In the 1990’s attempts were made to specifically target tumor characteristics with molecular imaging techniques based upon SPECT and PET technologies. The aim was to improve the accuracy of detecting PCa. Antibodies were initially developed, followed by peptides, to target PSMA as the basis for PCa specific molecular imaging agents.

The limitation in using intact antibodies for imaging is that clearance from circulation is quite slow and their maximum tumour uptake is reached at 3–7 days after injection; a fact that increases the non-specific target irradiation of healthy tissues. Small molecule PSMA-peptide inhibitor (also known as ligand) molecules have been developed which show high binding and these are the mainstay of current PSMA imaging techniques. Several PSMA ligands, differing slightly in chemical structure, are available. PSMA-HBED-CC (PSMA-11) is the most widely used ligand which can be easily labelled with ^68^Ga at room temperature with good stability and quick background clearance. However, there are some limitations with PET / CT ^68^Ga-PSMA: in fact they show a physiological intense uptake over salivary gland, liver, spleen, small bowel and urinary tract. Moreover the non specific absorption of radiotracers by non-cancerous bone remodeling processes can lead to false-positive results [22]. Furthermore, since ^68^Ga-PSMA is mainly excreted through the kidneys and accumulates in the urinary bladder, localization of small local recurrences adjacent to the bladder can be lost [23].

Imaging studies using smaller antibody fragments could be a good compromise between the long half life of the intact antibodies and the non specific binding of the peptides. The use of antibody fragments can accelerate clearance and improve rate of tumour penetration [24, 25] compared to the intact antibody allowing imaging the same or next day and preserving the antibody specificity.

The possibility to combine the specificity of small antibody formats with the high sensitivity and quantitative potential of PET to non-invasively identify cancer disease is the stepping stone to develop a powerful diagnostic tool.

Crucial to the growth of molecular imaging modalities is the continued development of radionuclide labeled targeting agents that can bind the target with high selectivity.

With the aim to overcome the limitation of radiopharmaceuticals currently used we choose to label the anti-PSMA scFvD2B with ^124^I. ^124^I has both beta radiation emissions and gamma emissions and according to its physical properties, ^124^I is the only long-life positron emitter isotope of iodine compatible with the half life of our antibody fragment; to achieve optimal tumour-to-background activity ratio in immuno-PET, it is indeed important to consider both the antibodies biological characteristics (specificity, kinetic of binding formats and half life) and the physical half life of the radionuclide [26].

We previously tested scFvD2B radiolabeled with ^131^I, ^111^In [27] and ^123^I [20] in preclinical in vivo models.

In particular, we can now consider in the same in vivo tumour model the T\B of the scFv D2B radiolabelled with 3 different iodine isotopes: the T/B of the ^131^I (half life 8.02 days) at 15 h and 24 h was 18 and 12 respectively [27], that of the ^123^I (half life 13.3 h) at 15 h and 24 h was 16 and 14 respectively [20] while the T/B of the ^124^I (half life 4.176 days) was around 40 both at 15 and 24 h.

Immuno-PET imaging data showed not only a significant uptake in PSMA-positive tumors 15 and 24 h after radiotracer administration but also a low accumulation in non-target organ and a rapid blood clearance.

Moreover the low background activity detected in the tumour’s surrounding tissue determines high-contrast PET images. The expected specificity of ^124^I- scFvD2B to reveal PCa was also confirmed by negligible accumulation in PSMA-negative tumours. The imaging analysis was confirmed by ex vivo biodistribution data obtained 15 and 24 h after radiotracer administration, supporting the hypothesis that ^124^I- scFvD2B could be an excellent radiopharmaceutical for PCa imaging.

These data indicate that using ^124^I it is possible to improve the radiotracer targeting.

Overall, the use of ^124^I-scFvD2B resulted in high-contrast immuno-PET images of PSMA-positive tumours with low accumulation in non-target organs probably due to the non residualizing properties of its radiocatabolites [28].

The indication for the use of imaging in prostate cancer is very important in all stages of disease: initial evaluation of disease extension, re-evaluation after primary treatments or in case of biochemical recurrence, appearance of resistance to hormones. The use of imaging therefore responds to different clinical questions of setting up initial treatments, guide for planning rescue therapies, assessing the effectiveness of a treatment. Current available imaging presents limitations related to the physiological biodistribution of the tracer with non-performing diagnostic performance in some disease settings.

The promising results for specific localization of the reagent and its low background absorption make immuno-PET an interesting clinical tool.

An optimal imaging that allows us to stage the disease, to see illnesses early and accurately after initial treatments, becomes increasingly important for setting personalized and correct therapeutic strategies.

However, as a basis for treatments it is necessary to have a precise detection of prostate cancer distribution. This is mandatory in order to select the most correct treatment for each patient.

Thanks to the accurate localization of disease, the possible use of immuno-PET therefore involves extensive diagnostic and therapeutic implications in clinical practice.

## Conclusions

The anti-PSMA scFvD2B has been shown to be stable and immunoreactive after labeling with ^124^I, a radiotracer suitable for PET imaging. In a preclinical in vivo model a specific localization of the reagent only in PSMA expressing tumors was obtained and excellent contrast images were provided. In translational perspective this immuno-PET reagent upon clinical confirmation of sensitivity and specificity can be a valuable tool to guide therapeutic strategies in PCa. Moreover our radiotracer could be used in human in a multi-modal systems such as PET/MR, PET/CT and PET/SPECT/CT changing the way in which cancer is treated, allowing a real personalized medicine approach.

## Data Availability

The data that support the findings of this study are available from the corresponding author upon reasonable request.

## Abbreviations

% ID/g: Percentage of injected dose per gram of tissue
BSA: Bovine serum albumin
CT: Computed tomography
FBS: Foetal bovine serum
FDG: Fludeoxyglucose
mAb: Monoclonal antibody
MRI: Magnetic resonance imaging
PBS: Phosphate Buffer Saline
PCa: Prostate Cancer
PET: Positron emission tomography
PSA: Prostate specific antigen
PSMA: Prostate-specific membrane antigen
scFv: Single-chain variable fragment
SPECT: Single photon emission computed tomography
STR: Short tandem repeat
T/B: Tissue/tumour to blood ratio
